# Effect of rising body temperature on respiratory chemosensitivity to CO_2_

**DOI:** 10.1186/2046-7648-4-S1-A152

**Published:** 2015-09-14

**Authors:** Keiji Hayashi, Takeshi Ogawa, Koji Sugiyama

**Affiliations:** 1 Junior College, University of Shizuoka, Shizuoka, Japan; 2 Osaka Kyoiku University, Osaka, Japan; 3 Faculty of Education, Shizuoka University, Shizuoka, Japan

## Introduction

A rise in body temperature (T_b_) is known to cause minute ventilation (V_E_) to increase. However, the mechanism of the ventilatory response to rising T_b _is still unclear. In the context of the relationship between V_E _and T_b_, it is known that respiratory chemosensitivity is influenced by T_b_, and that a rise in T_b _of more than 0.7 °C enhances respiratory chemosensitivity [1]. It is not known, however, whether increases in T_b _less than 0.7 °C also influence respiratory chemosensitivity. The aim of this study was to clarify the effect of mild hyperthermia (0.3 °C and 0.7 °C) on respiratory chemosensitivity.

## Methods

Eight persons (five males and three females, mean (SD) age 25 (10) years, height 171.5 (8.9) cm, weight 66.9 (8.7) kg) participated in the study. All were lowlanders and had not been exposed to altitude above 1,000 m within the 6 months prior the study. We measured sublingual temperature (T_sl_) as an index of T_b_, and measured respiratory chemosensitivity to CO_2 _using a rebreathing method [2]. The subjects wore a mask connected to a closed one-way circuit with a rubber bag containing the test gas (7 % CO_2_, 43 % O_2_, 50 % N_2_). Rebreathing was terminated when the inspired CO_2 _fraction reached 9.2 %. This test was performed before heating (ΔT_sl _= 0 °C) and during heating (ΔT_sl _= 0.3 °C and 0.7 °C). Measurements were made twice with a 15-min interval between tests at ΔT_sl _= 0°C, 0.3°C and 0.7°C. During the experiment subjects wore a water-perfused suit. The initial water temperature was 35 °C and was increased to 45 °C.

## Results

Before heating mean (SD) T_sl _was 36.15 (0.22) °C (ΔT_sl _= 0 °C) and rose to 36.47 (0.21) °C at ΔT_sl _= 0.3 °C and then to 36.87 (0.21) °C at ΔT_sl _= 0.7 °C during heating. While subjects breathed the CO_2_-rich mixture, V_E _was 1.49 (0.68) L.min^-1^.mmHg^-1 ^(ΔT_sl _= 0 °C), 1.52 (0.75) L.min^-1^.mmHg^-1 ^(ΔT_sl _= 0.3°C) and 1.75 ± 0.98 L.min^-1^.mmHg^-1 ^(ΔT_sl _= 0.7°C). The tidal volume was 44.7 (12.4) mL.mmHg^-1 ^(ΔT_sl _= 0°C), 55.4 (24.9) mL.mmHg^-1 ^(ΔT_sl _= 0.3 °C) and 61.9 (19.5) mL.mmHg^-1 ^(ΔT_sl _= 0.7 °C) (P < 0.06). The respiratory frequency was 0.47 (0.38) breaths.min^-1^.mmHg^-1 ^(ΔT_sl _= 0 °C), 0.40 (0.42) breaths.min^-1^.mmHg^-1 ^(ΔT_sl _= 0.3 °C) and 0.37 (0.41) breaths.min^-1^.mmHg^-1 ^(ΔT_sl _= 0.7 °C).

## Discussion

These results suggest that increases in T_sl _less than 0.7 °C do not influence respiratory chemosensitivity to CO_2_, though the respiratory pattern did tend to change. The ventilatory response to rising T_b _has a threshold around 38 °C (esophageal temperature) in the resting state [3]. Moreover, we suggest that increasing the inspired CO_2 _fraction did not reduce that threshold to the temperatures reached in the present study (T_sl _around 37 °C).

## Conclusion

Our findings suggest that respiratory chemosensitivity is not affected by mild hyperthermia (~0.7 °C rise in body temperature). It is possible that there is a T_b _threshold for changes in respiratory chemosensitivity that is greater than around 37 °C.
